# Vitamin D_3_ metabolite ratio as an indicator of vitamin D status and its association with diabetes complications

**DOI:** 10.1186/s12902-020-00641-1

**Published:** 2020-10-27

**Authors:** Lina H. M. Ahmed, Alexandra E. Butler, Soha R. Dargham, Aishah Latif, Omar M. Chidiac, Stephen L. Atkin, Charbel Abi Khalil

**Affiliations:** 1 Weill Cornell Medicine-Qatar, PO Box 24144, Doha, Qatar; 2 Diabetes Research Center (DRC), Qatar Biomedical Research Institute (QBRI), Hamad Bin Khalifa University (HBKU), Qatar Foundation (QF), PO Box 34110, Doha, Qatar; 3 AntiDoping Lab Qatar, Doha, Qatar; 4 Royal College of Surgeons of Ireland, Manama, Bahrain

**Keywords:** Vitamin D, Vitamin D metabolites, Vitamin D deficiency, Vitamin D metabolite ratio, Diabetic complications

## Abstract

**Background:**

Vitamin D deficiency is diagnosed by total serum 25-hydroxyvitamin D (25(OH)D) concentration and is associated with poor health and increased mortality; however, some populations have low 25(OH) D concentrations without manifestations of vitamin D deficiency. The Vitamin D Metabolite Ratio (VMR) has been suggested as a superior indicator of vitamin D status. Therefore, VMR was determined in a population with type 2 diabetes at high risk for vitamin D deficiency and correlated with diabetic complications.

**Research design and methods:**

Four hundred sisty patients with type 2 diabetes (T2D) were recruited, all were vitamin D_3_ supplement naive. Plasma concentration of 25-hydroxyvitamin D_3_ (25(OH)D_3_) and its metabolites 1,25-dihydroxyvitamin D_3_ (1,25(OH)_2_D_3_) and 24,25-dihydroxyvitamin D_3_ (24,25(OH)_2_D_3_) and its epimer, 3-epi-25-hydroxyvitamin D_3_ (3-epi-25(OH)D_3_), were measured by LC-MS/MS analysis. VMR-1 was calculated as a ratio of 24,25(OH)_2_D_3_:25(OH)D_3_; VMR-2 as a ratio of 1,25(OH)_2_D_3_:25(OH)D_3_; VMR-3 was calculated as a ratio of 3-epi-25(OH)D_3_: 25(OH)D_3._

**Results:**

An association means that there were significant differences between the ratios found for those with versus those without the various diabetic complications studied. VMR-1 was associated with diabetic retinopathy (*p* = 0.001) and peripheral artery disease (*p* = 0.012); VMR-2 associated with hypertension (*p* < 0.001), dyslipidemia (*p* < 0.001), diabetic retinopathy (*p* < 0.001), diabetic neuropathy (*p* < 0.001), coronary artery disease (*p* = 0.001) and stroke (*p* < 0.05). VMR-3 associated with hypertension (*p* < 0.05), dyslipidemia (*p* < 0.001) and coronary artery disease (*p* < 0.05).

**Conclusions:**

In this cross sectional study, whilst not causal, VMR-2 was shown to be the superior predictor of diabetic and cardiovascular complications though not demonstrative of causality in this cross-sectional study population over VMR-1, VMR-3 and the individual vitamin D concentration measurements; VMR-2 associated with both microvascular and cardiovascular indices and therefore may have utility in predicting the development of diabetic complications.

**Supplementary Information:**

**Supplementary information** accompanies this paper at 10.1186/s12902-020-00641-1.

## Background

**Fig. 1:**
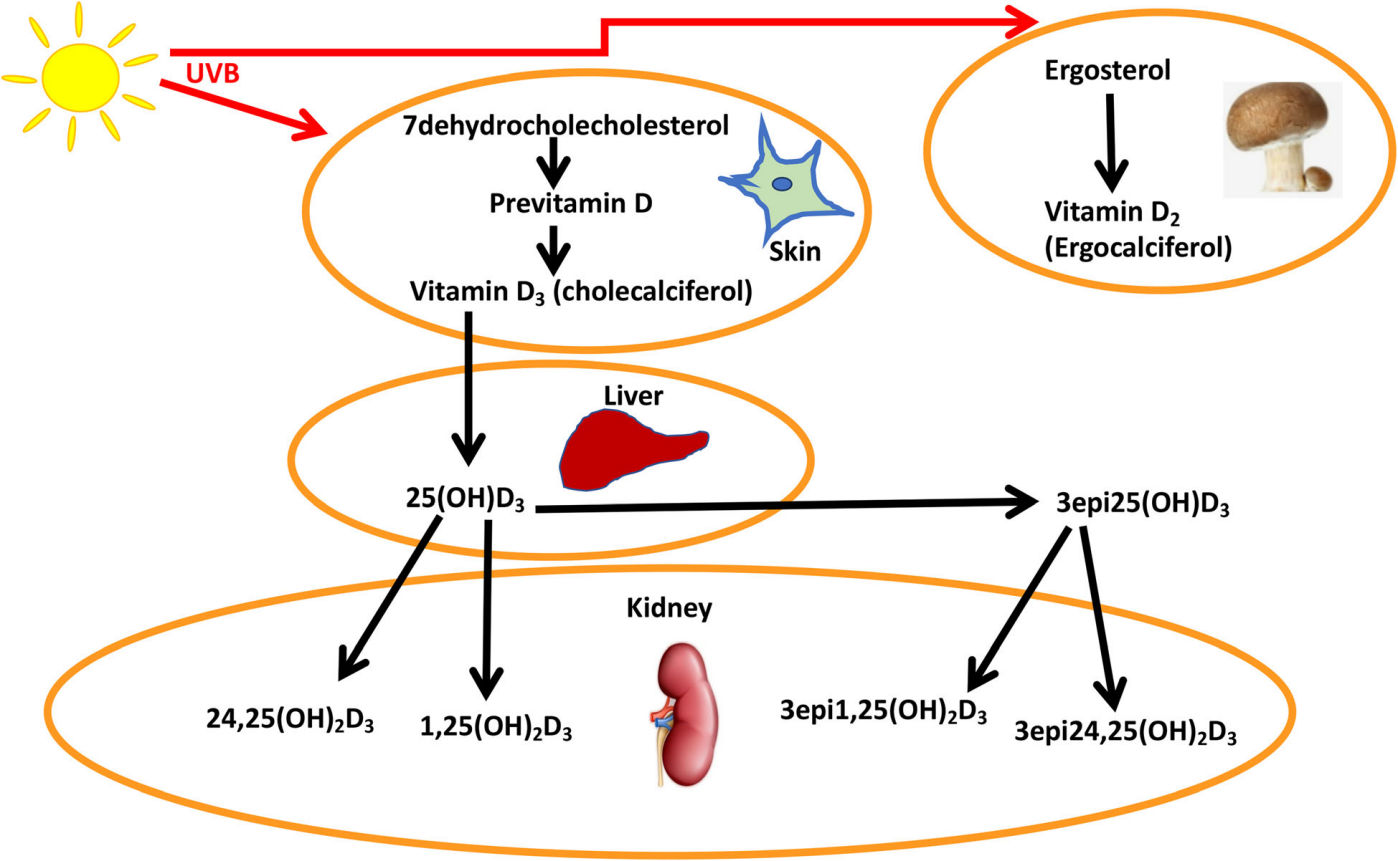
The Vitamin D_3_ pathway. In the skin, 7-dehydrocholesterol is converted to previtamin D_3_ and then to vitamin D_3_. This is transported to the liver, converted to 25 hydroxyvitamin D (25(OH)D_3_) and then transported to the kidney. In the kidney, 25(OH)D_3_ undergoes conversion to the active 1,25 (OH)_2_D_3_, and 24,25(OH)_2_D_3_

Vitamin D comprises a group of fat-soluble steroids, vitamin D_3_ (cholecalciferol) and vitamin D_2_ (ergocalciferol) being the two major compounds in humans. Vitamin D’s major role is facilitation of intestinal absorption of calcium, magnesium and phosphate, and it is therefore central to calcium homeostasis and bone metabolism [1]. Whilst some foods such as mushrooms and fungi contain vitamin D_2_ [2], most vitamin D (Vitamin D_3_ (25(OH)D_3_)) is derived from conversion of cholesterol to cholecalciferol in the skin, a process activated by UVB radiation from sunlight exposure. 25(OH)D_3_ is inert and must undergo hydroxylation in the kidney to its active form, 1,25 dihydroxyvitaminD_3_ (1,25(OH)D_3_ [3] or to 24,25-dihydroxyvitamin D (24,25(OH)_2_D3) by 24 alpha hydroxylase in the renal tubular cells (Fig. 1) [4]. While vitamin D deficiency represents a worldwide health issue [5], it is exacerbated in some parts of the world, such as the Middle East, where a notably high prevalence is consequent upon local cultural norms requiring full body coverage [6–9].

Vitamin D deficiency has been associated with a range of negative health outcomes, including osteoporosis and type 2 diabetes, as well as increased mortality that may be addressed by vitamin D supplementation [10]. Diagnosis is widely based upon measurement of total serum 25-hydroxyvitamin D (vitamin D_2_ plus vitamin D_3_), a value of < 20 ng/ml (< 48.4 nmol/l) being indicative of vitamin D insufficiency. However, certain ethnic groups appear to have low serum concentrations of 25(OH) D whilst maintaining healthy bone mineral density. African Americans represent one such group, with typically low concentrations of 25(OH) D [11–13] and yet with a higher bone mineral density and a lower risk of osteoporosis and fractures than their white counterparts [14–17]. 1,25-dihydroxyvitamin D concentrations (1,25(OH)_2_D) are related not only to kidney function but also to vitamin D status, and patients who are vitamin D deficient or insufficient have normal or even high concentrations of 1,25(OH)_2_D due to secondary hyperparathyroidism [3]. 24,25(OH)_2_D is not only related to the blood concentrations of 25-hydroxyvitamin D but are also related to the blood concentrations of 1,25(OH)_2_D because it induces 24 hydroxylase [3]. Whilst 1,25(OH)_2_D is the active metabolite of 25(OH) D, 24,25(OH)_2_D_3_ is also an active metabolite (it can be converted to 1,24,25-trihydroxyvitamin D_3_ through the C24 oxidation pathway [18]) as it has been shown to induce non-genomic signalling pathways, a mechanism active in many tissues, playing, for example, a physiological role in growth plate formation [3] and in activating rapid insulin release from pancreatic beta cells in response to increases in glycemia [19].

3- epimerase isomerizes the C-3 hydroxy group of 25(OH) D from the α to the β orientation leading to 3epi25(OH) D [3, 20] that may be measured inadvertently whilst measuring 25(OH) D [21]. 3epi25(OH) D is thought to be less potent physiologically when compared with 25(OH) D and 1,25(OH)_2_–3-epi-D; however, whilst data is sparse on the biological potency and role of the C3 epimers, we have reported that it was not associated with diabetes complications [22].

We have previously shown that type 2 diabetes (T2D) complications are associated with differing metabolites of vitamin D: diabetic retinopathy associated with lower 25(OH)D_3_ and 1,25(OH)_2_D_3_ concentrations; hypertension associated with lower 1,25(OH)_2_D_3_, and dyslipidemia associated with lower 25(OH)D_3,_ 1,25(OH)_2_D_3_ and 24,25(OH)_2_D_3_ [22]_._ The vitamin D metabolite ratio (VMR), a ratio of 24,25(OH)_2_D: 25(OH) D, has been proposed at a better indicator of vitamin D status [23]. This is particularly important in ethnic groups where low 25(OH) D is prevalent, as VMR can identify individuals who are functionally deficient from those who are not. We therefore sought to determine the following three vitamin D metabolite ratios: 24,25(OH)_2_D_3_: 25(OH)D_3_ (termed VMR-1), 1,25(OH)_2_D_3_: 25(OH)D_3_ (termed VMR-2) and 3-epi-25(OH)D_3_: 25(OH)D_3_ (termed VMR-3) in a cohort of Middle Eastern type 2 diabetic subjects with normal renal function where low 25(OH)D_3_ is the norm.

## Research design and methods

### Study population

460 Middle Eastern type 2 diabetic subjects were recruited from June 2012–2013 from the Hamad outpatient clinic, Doha, Qatar as part of a study designed primarily to investigate gene expression and genomics in diabetic subjects (Table 1) [24].

**Table 1:** Demographic data, Vitamin D_3_ levels and Vitamin D_3_ Metabolite Ratios (VMR) for Type 2 Diabetes (*n* = 460) patients

	Type 2 Diabetes ***n*** = 460
**Age (years)** **mean (SD)**	55.2 (9.9)
**Male Gender** **N (%)**	227 (49.4)
**BMI (kg/m**^**2**^**)** **median (IQR)**	32.4 (28.6–37.2)
**HbA1c (%)** **median (IQR)**	7.9 (6.7–9.5)
**Glucose (mmol/l)** **median (IQR)**	8.6 (6.4–12.2)
**1,25(OH)**_**2**_**D**_**3**_ **(ng/dl)** **median (IQR)**	0.02 (0.01–0.04)
**25(OH)D**_**3**_ **(ng/dl)** **median (IQR)**	6.5 (3.4–13.6)
**24,25(OH)**_**2**_**D**_**3**_ **(ng/dl)** **median (range)**	0.3 (0.2–0.6)
**3-epi-25(OH)D**_**3**_ **median (IQR)**	0.4 (0.2–0.8)
**VMR-1 [24,25(OH)**_**2**_**D**_**3**_**:25(OH)D**_**3**_**] median (IQR)**	0.05 (0.04–0.07)
**VMR-2 [1,25(OH)2D3:25(OH)D3] median (IQR)**	0.002 (0.001–0.004)
**VMR-3 [3-epi-25(OH)D3:25(OH)D3 median (IQR)**	0.07 (0.05–0.10)

*1,25(OH)*_*2*_*D*_*3*_ 1,25-Dihydroxyvitamin D_3_; *25(OH)D*_*3*_ 25-hydroxyvitamin D_3_; *24,25(OH)*_*2*_*D*_*3*_ 24,25-dihydroxyvitamin D_3_; *3-epi-25(OH)D*_*3*_ 3-epi-25-hydroxyvitamin D_3;_ *IQR* Interquartile range

Males or females aged 30 years or older were included in the study; all had normal renal function and none were taking vitamin D_3_ supplements. A diagnosis of T2D was based upon WHO guidelines [25] with one or more of the following criteria: fasting plasma glucose > 7 mmol/l, HbA1c > 6.5%, or a diagnostic glucose tolerance test. Exclusion criteria were a diagnosis of type 1 diabetes or secondary diabetes, such as gestational diabetes or that due to steroid treatment.

The study was approved by Weill Cornell IRB (IRB# 13–00063) and all participants provided written informed consent. The conduct of this study was in accordance with ICH GCP and the Declaration of Helsinki.

### Study design

At the baseline visit, blood samples were collected following an overnight fast and weight and blood pressure were measured. Blood pressure measurement was standardised with the non-smoking patient in a seated position, resting quietly for 5 min prior to the first reading. The arm was supported with the elbow at the level of the heart. Readings were taken 3 times, and the lowest reading was selected for analysis. A wide cuff sphygmomanometer was used in obese patients. Fasting venous blood was collected into fluoride oxalate and serum gel tubes. Samples were centrifuged at 2000 *g* for 15 min at 4 °C, with aliquots stored at − 80 °C within 1 h of collection. Blood pressure was measured with an automated device (NPB-3900; Nellcor Puritan Bennett, Pleasanton, CA) at each study visit.

### Serum vitamin D_3_ measurement

Measurement of vitamin D and its metabolites have previously been described in detail [22]. In brief, serum vitamin D_3_ concentrations were quantified using isotope-dilution liquid chromatography tandem mass spectrometry (LC-MS/MS). “25 μL of internal standards (d6-1calcitriol (1.5 ng/mL), d6-25OHD_3_ (50 ng/mL) and d6-epi-25(OH)D_3_ (20 ng/mL)) were added into each microcentrifuge tube containing 250 μL of calibration standards, Quality Control or serum samples, and kept for 30 min to reach binding equilibrium. The samples were diluted with 250 μL of pretreatment solution (isopropanol and water; 50:50 v/v) and left to stand for at least 15 min to displace binding protein.

300 μL of pre-treated samples were loaded onto ISOLUTE® supported liquid extraction (SLE+) columns (Biotage), followed by elution with 1.8 mL of n-heptane (2 × 900 μL) into a collection tube already containing 200 μL of 0.25 mg/mL PTAD solution in ethyl acetate and heptane (8:92 v/v). The eluate was evaporated to dryness using turbovap under nitrogen gas heated at 38 °C. Once dried, 50 μL of reconstituted solution consisting of methanol and deionized water, 70:30 v/v, and 0.006% methylalamine were added into all tubes. The derivatized extracts were transferred into LC insert vials and 10 μL from each was injected into the LC-MS/MS system. Data for the 25(OH)D_3_ and metabolite validation is shown in Supplementary Table 1.”

### Study outcomes

#### Statistical analyses

Data trends were visually and statistically evaluated for normality. A Student’s t-test was used for normally distributed data; when those data were not normally distributed, then the Kolmogorov-Smirnov Test and non-parametric tests (Mann Whitney U) were utilised. Statistical analysis was performed using SPSS for Windows, version 24.0. All values are given as mean ± SD or as mean with 95% confidence interval (CI) unless specified.

## Results

The baseline characteristics, including the demographics, for the type 2 diabetes patients are shown in Table 1.

subjects. The relationship of Vitamin D_3_ Metabolite Ratios (VMRs) with diabetes complications in this cohort (*n* = 460) of subjects with Type 2 Diabetes is shown in Table 2.

**Table 2:**
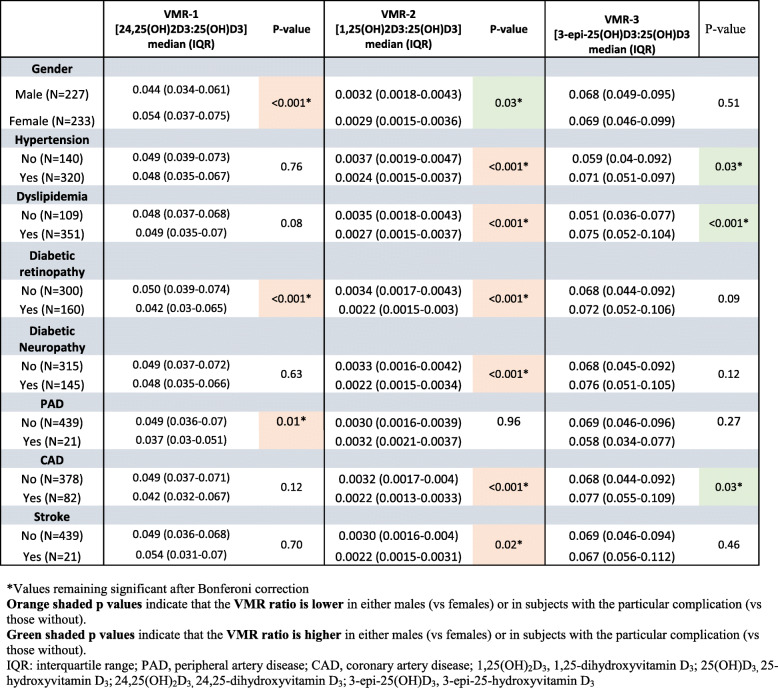
Relationship of Vitamin D_3_ Metabolite Ratios (VMR) with diabetes complications in the cohort (*n* = 460) of subjects with Type 2 Diabetes

As we have previously reported, concentrations of 25(OH)D_3,_ 1,25(OH)D_3_, 24,25(OH)_2_D_3_ and 3epi-25(OH)D_3_ were all lower in females (*p* = 0.003); however, despite the lower vitamin D concentrations measured in females, there was no difference in diabetes complication rates between males and females [22].

An association here means that there was a significant difference between the ratios found in those with versus without the diabetic complications discussed. The VMR-2 ratio showed a striking association with diabetic complications, namely hypertension (*p* < 0.001), dyslipidemia (*p* < 0.001), diabetic retinopathy (*p* < 0.001), diabetic neuropathy (*p* < 0.001), coronary artery disease (*p* < 0.001) and stroke (*p* < 0.018). By way of comparison, 25(OH)D_3_ associated with dyslipidaemia (*p* < 0.04) and diabetic retinopathy (*p* < 0.03), while 1,25(OH)D_3_ alone was associated with hypertension (*p* < 0.009), dyslipidaemia (*p* < 0.003), retinopathy (*p* < 0.006) and coronary artery disease (*p* = 0.012), as we have previously reported [22].

The VMR-1 ratio showed a relationship with diabetic retinopathy (*p* = 0.001) and peripheral artery disease (*p* = 0.012) that was not revealed using 24,25(OH)_2_D_3_ concentrations alone [22].

The VMR-3 ratio showed a relationship with hypertension (*p* = 0.03), dyslipidaemia (*p* < 0.001) and coronary artery disease (*p* = 0.034). For comparison, 3epi-25(OH)D_3_ alone associated only with diabetic neuropathy, as previously reported (*p* = 0.03) [22].

In view of the potential confounding influence of renal function, estimated glomerular filtration rate (eGFR) was correlated to vitamin D_3_ and its metabolites. The normal range for eGFR is 100–130 mL/min/1.73m^2^ in men and 90–120 mL/min/1.73m^2^ in women below the age of 40 years. eGFR decreases with age, decreasing to a mean of 99 mL/min/1.73m^2^ in the 40–49 years age range, 93 mL/min/1.73m^2^ from 50 to 59 years and 85 mL/min/1.73m^2^ from 60 to 69 years. All the subjects in this study had eGFR in the normal range for age and gender. The only correlation with eGFR was found with 24,25(OH)_2_D_3_ (1,25(OH)_2_D_3_: *R* = 0.067 *p* = 0.24; 25(OH)D_3_: *R* = -0.032, *p* = 0.51; 24,25(OH)_2_D_3_: *R* = 0.148, *p* = 0.002).

The effect of age and duration of diabetes on the VMR ratios and each of the reported complications was also considered. Age did influence VMR-2 (*r*^*2*^ = − 0.26, *p* < 0.001) but had no influence on VMR-1 (*r*^*2*^ = 0.004, *p* = 0.93) or VMR-3 (*r*^*2*^ = 0.07, *p* = 0.21). Duration of diabetes had an effect on all three VMRs (VMR-1: *r*^*2*^ = − 0.14, *p* = 0.004; VMR-2: *r*^*2*^ = − 0.22, *p* < 0.001; VMR-3: *r*^*2*^ = 0.14, *p* = 0.011).

There was a relationship of age with each of the following complications: hypertension (*p* < 0.001), dyslipidemia (*p* < 0.001), diabetic retinopathy (*p* = 0.001), diabetic neuropathy (*p* < 0.001), CAD (*p* < 0.001) and stroke (*p* = 0.03), the only complication not showing a relationship with age being PAD (*p* = 0.98) (Table 3). Likewise diabetes duration showed a relationship with the same complications as age: hypertension (*p* < 0.001), dyslipidemia (*p* < 0.001), diabetic retinopathy (*p* < 0.001), diabetic neuropathy (*p* < 0.001), CAD (*p* < 0.001) and stroke (*p* = 0.001); again, the only complication not showing a relationship with diabetes duration being PAD (*p* = 0.18) (Table 3).

**Table 3:**
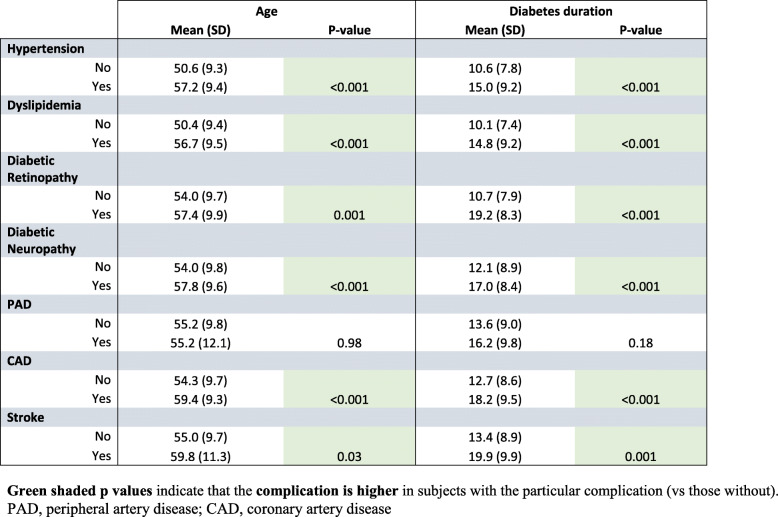
Relationship of age and diabetes duration with diabetes complications in the cohort (*n* = 460) of subjects with Type 2 Diabetes. The data is normally distributed so the mean and SD have been reported

## Discussion

The VMR-2 ratio showed a striking relationship between diabetic complications being lower in diabetic retinopathy and diabetic neuropathy, and the cardiovascular complications of hypertension, dyslipidemia, coronary artery disease and stroke. These findings suggest that the VMR-2 ratio is a superior predictor for development of diabetic and cardiovascular complications. We have previously reported the association of diabetic microvascular complications with 1,25(OH)_2_D_3_, 25(OH)D_3_ and its epimers [22]. In this current study, we hypothesized that an alternative VMR ratio (termed VMR-2) comparing 1,25(OH)_2_D_3_ to 25(OH)D_3_ may have even greater predictive power for diabetic and cardiovascular complications, as 1,25(OH)_2_D_3_ is the active metabolite of vitamin D_3_ (25(OH)D_3_) [3]. It should also be noted that 1,25(OH)_2_D_3_ to 25(OH)D_3_ appeared to be independent of the estimated glomerular function and therefore VMR-2 was unaffected. Here, it should be noted that serum calcitriol concentration is tightly regulated and would not be expected to vary when renal function is in the normal range, as is the case in this study cohort [26]. Further, all target tissues activate vitamin D, where it has paracrine and autocrine functions locally, but whether such calcitriol enters the circulation is unclear [27, 28].

The VMR-1 ratio was less discriminatory than VMR-2, associating only with diabetic retinopathy and peripheral artery disease and, in addition, the 24,25(OH)_2_D_3_ correlated to the eGFR, suggesting that this VMR-1 ratio would be affected by renal function. The VMR-3 ratio did not prove to be a usefully discriminatory measure, though it did associate with more complications than 3epi-25(OH)D_3_ alone.

This Middle East population is an ethnic group at high risk for vitamin D deficiency with all the associated negative outcomes such as increased risks of bone disease as well as diabetic and cardiovascular complications [5, 8, 9]. However, given the fact that the circulating concentrations of 25-hydroxyvitamin D_3_ (25(OH)D_3_) are universally low in this population [8], a measure of vitamin D status that could distinguish healthy individuals despite their having a low 25(OH)D_3_ versus individuals with low 25(OH)D_3_ at high risk for development of diabetic and cardiovascular complications, would be clinically useful.

When compared with Caucasian Americans, African Americans tend to have lower concentrations of 25(OH) D [11–13], often meeting the criteria for vitamin D insufficiency, and yet have more robust bone health [14–17]. Therefore, 25(OH) D alone is not always a discriminatory test depending on the population group. The metabolite of 25(OH) D, 24,25(OH)_2_D, has been proposed as an additional useful measure for several reasons [29, 30]. Firstly, concentrations of 24,25(OH)_2_D and 25(OH) D are closely correlated [31]. Secondly, 25(OH) D is converted to 24,25(OH)_2_D by CYP24A1, a 24-hydroxylase enzyme which is partially regulated by vitamin D receptor activity [32] [33]. However, the VMR-1 ratio was not found to be of greater value than VMR-2 for predicting risk of diabetic and cardiovascular complications.

Strengths of this study are the well-characterized, homogeneous Middle East population with well-recognized vitamin D deficiency, and that vitamin D_3_ and its metabolites were measured on state-of-the-art equipment. Limitations of this study include the fact that it was a cross sectional design and the relatively modest numbers of subjects for such a population-based study, but this limitation is mitigated by the homogeneous nature of the population studied. A prospective study would be necessary to validate the results found here. Furthermore, our findings here may not be applicable to other ethnic groups or countries, since Middle Easterners have low vitamin D status, in part, because of their primarily vegetable-based diet, near total skin coverage and tendency to stay indoors to avoid the hot summer sun [34].

## Conclusion

In conclusion, in type 2 diabetes, VMR-2 was shown to be the superior predictor for development of diabetic and cardiovascular complications in this study population over VMR-1, VMR-3 and the individual vitamin D concentration measurements, associating with both microvascular and cardiovascular indices and therefore may have utility in predicting the development of diabetic complications.

## Supplementary Information

**Additional file 1.**

## Data Availability

All data underlying this study will be made available upon reasonable request to the corresponding author.

## Abbreviations

VMR: Vitamin D Metabolite Ratio
25(OH)D3: 25-hydroxyvitamin D3
1,25(OH)2D3: 1,25-dihydroxyvitamin D3
24,25(OH)2D3: 24,25-dihydroxyvitamin D3
3-epi-25(OH)D3: 3-epi-25-hydroxyvitamin D3
T2D: Type 2 Diabetes
LC: liquid chromatography
LC-MS/MS: Liquid chromatography-mass spectrometry/mass spectrometry
HbA1c: glycosylated hemoglobin
WHO: World Health Organization
IRB: Internal Review Board
ICH GCP: International Conference on Harmonization Good Clinical Practice
SLE: supported liquid extraction
PTAD: 4-(4-(2-Azidoethoxy)phenyl)-1,2,4-triazolidine-3,5-dione
SPSS: Statistical Package for the Social Sciences
eGFR: estimated glomerular filtration rate
CAD: coronary artery disease
PAD: peripheral artery disease
CYP24A1: Cytochrome P450 family 24 subfamily A member 1
