# Supplementary material for: Vitamin D3 metabolite ratio as an indicator of vitamin D status and its association with diabetes complications
Source: BMC Endocr Disord. 2020 Oct 27;20:161. doi: 10.1186/s12902-020-00641-1 (PMC7590744; doi:10.1186/s12902-020-00641-1)
Supplement: Supplementary file 1 — Additional file 1. [file 12902_2020_641_MOESM1_ESM.docx]

**Supplementary Table 1.** **Validation of Vitamin D metabolite measurements**.

Analyses for all 8 Vitamin D metabolites were validated for LOQ, linearity, accuracy, repeatability, reproducibility and robustness. All analytes showed good recoveries with acceptable accuracy and within ±15% reproducibility.

| **Vitamin D Metabolites** | **LOQ** | **Linearity Range** | **Accuracy** | **Reproducibility CV%** |
| --- | --- | --- | --- | --- |
| 25(OH)D_3_ | 0.5 ng/mL | 0.5-100 ng/mL | 102-118% | 6.8 |
| 24-R-25(OH)_2_D_3_ | 0.05 ng/mL | 0.05-10 ng/mL | 94-102% | 5.7 |
| 1,25(OH)_2_D_3_ | 0.01 ng/mL | 0.01-0.20 ng/mL | 89-105% | 12.3 |
| 3-epi-25(OH)D_3_ | 0.05 ng/mL | 0.05-10 ng/mL | 67-106% | 13.7 |

LOQ, limit of quantification; CV, coefficient of variation.
